# Abdomen cocoon causing chronic abdominal pain and intestinal obstruction; a case series

**DOI:** 10.1016/j.amsu.2019.10.003

**Published:** 2019-10-15

**Authors:** Sardar Hassan Arif, Ayad Ahmad Mohammed

**Affiliations:** Department of Surgery, College of Medicine, University of Duhok, Kurdistan Region, Iraq

**Keywords:** Cocoon abdomen, Intestinal obstruction, Sclerosing encapsulating peritonitis, Peritoneal encapsulation

## Abstract

Abdomen cocoon is a rare disease in which a thick peritoneal membrane wraps the intestine causing the bowel loops to adhere to each other. It may be either primary(idiopathic) or secondary to other causes like previous abdominal surgery. Most patients present with abdominal pain and intestinal obstruction. The condition is usually diagnosed intraoperatively.

**Case 1:**

A 30-year-old male patient presented with abdominal pain and bilious vomiting. The patient had similar previous attacks. Examination showed distension abdominal distension with central tenderness. Plain abdominal X-ray showed multiple air fluid levels. During surgery most of ileum was enclosed by thin membrane with dilated proximal jejunum. Release of the bowel loops was done. The patient was well after surgery and was discharged with no post-operative complications.

**Case 2:**

A 35-year old male presented with chronic right lower quadrant abdominal pain, the past medical and surgical histories were non-relevant. Abdominal examination showed tenderness on deep palpation at the right iliac fossa, abdominal ultrasound and abdominal X-ray were normal. During diagnostic laparoscopy the terminal ileum was enclosed with a thick whitish membrane with dilated proximal ileum. Release of the adhesions was done. The patient was well in the post-operative period and he was discharged home with no post-operative complications.

In both cases the biopsy from the membranes showed features of chronic inflammatory process.

Abdomen cocoon is one of the rare causes of small bowel obstruction. The bowel adhesions should be opened and nonviable segments resected. Most patients have good long term outcome.

## Highlights

• Abdomen cocoon is a rare disease in which a thick membrane wraps the intestine.
• It is either primary(idiopathic) or secondary to previous abdominal surgery.
• Most patients present with abdominal pain and intestinal obstruction which is usually diagnosed intraoperatively.

## 1 Introduction

Abdomen cocoon or called sclerosing encapsulating peritonitis is a rare disease that particularly affects women and is very rare in men. In this condition a thick peritoneal membrane wraps the intestine causing the bowel loops to adhere to each other. A variable length of the bowel may be affected [[1], [2], [3], [4]].

This condition was first described by Foo et al., in 1978, when he presented a series of 6 girls who presented with intestinal obstruction, the cases were encountered over a period of 6 years, all were young females between 13 and 18 years of age and he described the condition to occur within two years of menarche suggesting a possible link. Foo grouped their medical condition under a clinical entity named as “the abdominal cocoon”, later this presentation drew the attention of surgeons from different parts of the world to similar cases and they started to document them and presented many cases and articles, and many possible cause were described [5].

The etiology of this condition is unclear but histopathological examination shows features of chronic inflammation. It may be either primary(idiopathic) or secondary which is seen in association with some conditions such as previous abdominal surgery, complicated abdominal wall hernia, renal and liver transplantation, peritoneo-venous shunt in liver cirrhosis [1,3,6,7].

Most patients present with abdominal pain and intestinal obstruction which is usually diagnosed intraoperatively. CT-scan or barium study can help in suspecting the condition before surgery [1,2,8].

The work of this case report has been reported in line with the PROCESS 2018 criteria [9].

## 2 Methods

This is a retrospective case series that includes two patients who were presented with chronic undiagnosed abdominal pain and one with intestinal obstruction were included.

In one patient open surgery was done and the other one diagnostic laparoscopy were done.

The operations were done by two general surgeons who are specialized in the field of general surgery and minimally invasive surgery. The operations were done Duhok Emergency Teaching Hospital and in Azadi Teaching Hospital, which are two academic institutions. The two cases were encountered within a period of 3 years.

This case series is registered according to the World Medical Association's Declaration of Helsinki 2013 at the research registry in the 14th of August 2019; the unique identifying number is: researchregistry5 080.

## 3 Results

### 3.1 Case 1

A 30-year-old male patient presented to the emergency hospital with colicky central abdominal pain for 12 hours, the pain was associated with 2 attacks of bilious vomiting. The patient has history of similar attacks of milder intensity of pain before 1 year which were managed conservatively. There was not relevant past surgical history and the medical history was negative for comorbid illnesses.

The pulse rate was 100 beats/minute, the blood pressure was 130/70 mm Hg and the patient was a febrile. Examination of the abdomen showed symmetrical distension of the abdomen with central tenderness and exaggerated bowel sound.

**Fig. 1:**
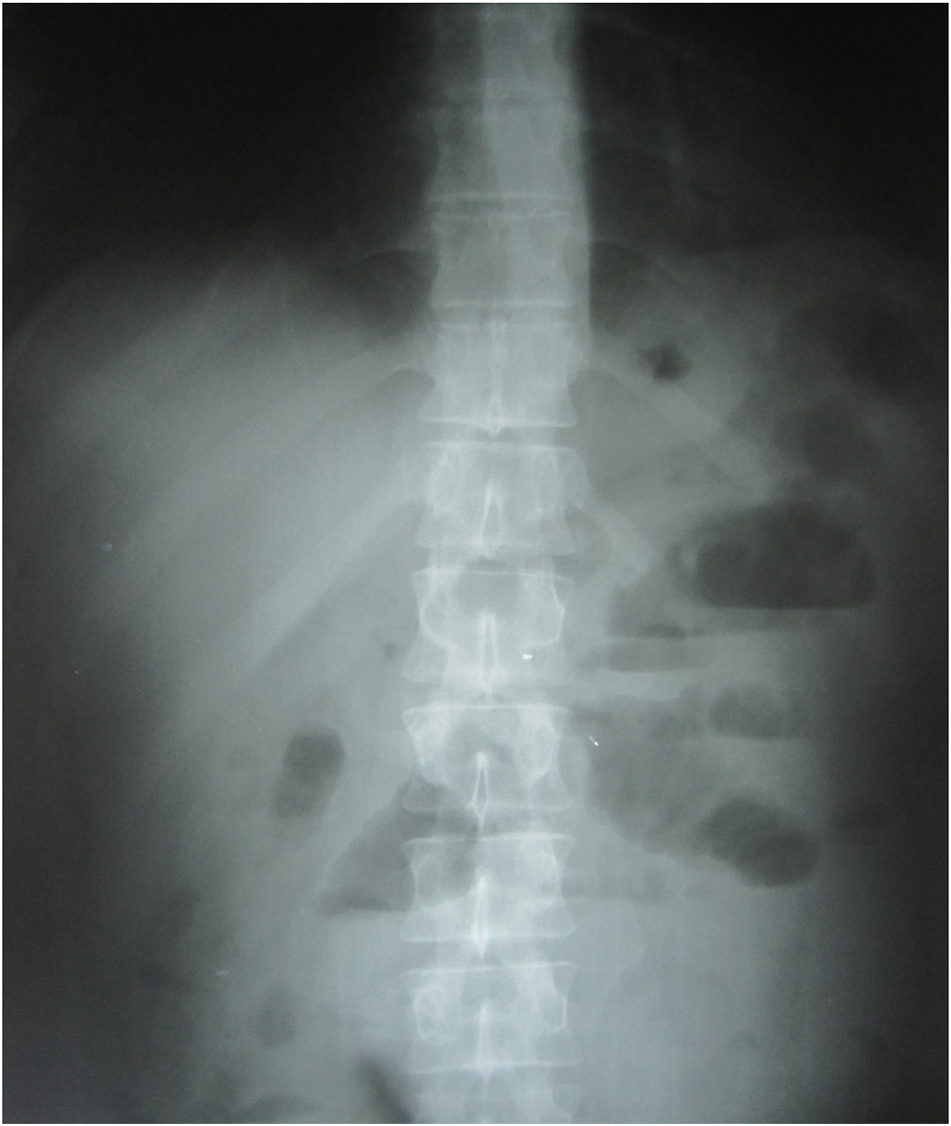
Plain abdominal X-ray in erect position showing multiple air-fluid levels suggesting intestinal obstruction.

Plain abdominal X-ray in erect position showed multiple air fluid levels in the center of the abdomen. Fig. 1.

**Fig. 2:**
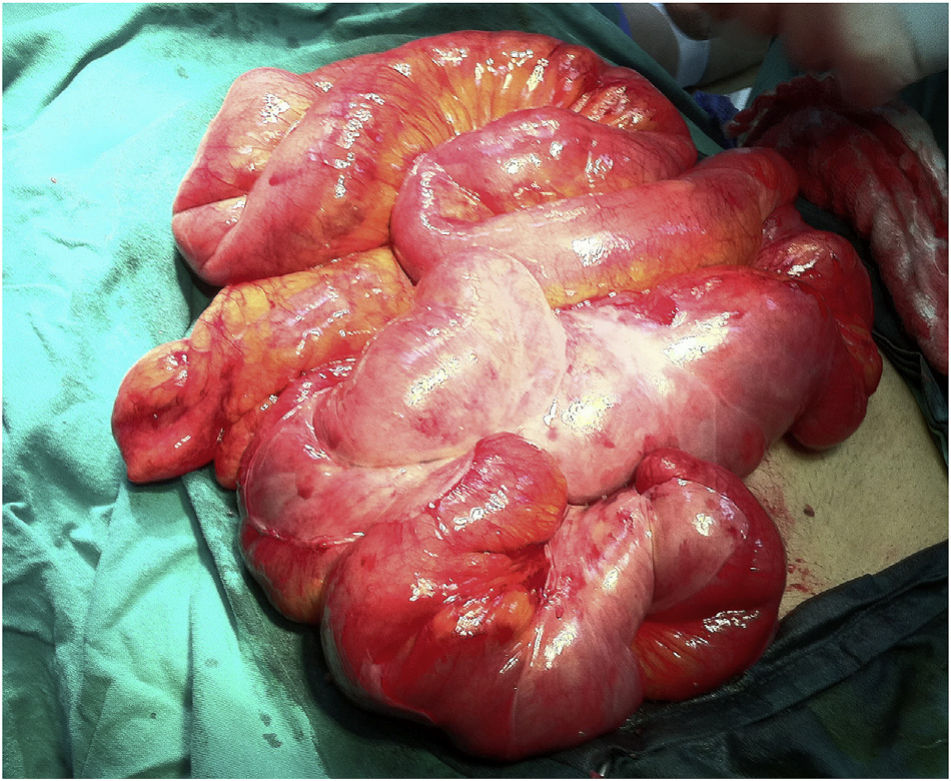
Intraoperative picture showing the terminal part of the small bowel is enclosed in a membrane like layer with dilatation of the proximal bowel.

During surgery through right lower para-median incision, most of ileum was found to be enclosed by thin membrane with dilated proximal jejunum. Fig. 2.

**Fig. 3:**
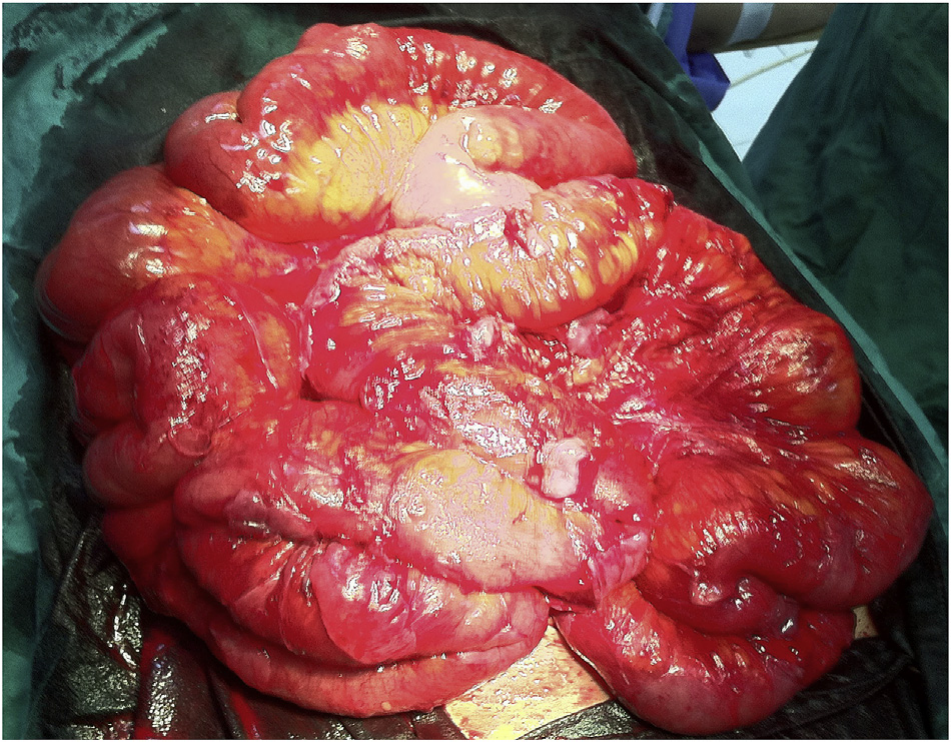
Intraoperative picture showing the small bowel after being released completely.

Release of the bowel loops was done by cutting the membrane in between and all the adhesions were opened, the bowel was viable and no resection was required. A piece of the membrane was sent for histopathological study which showed features of chronic inflammatory process with no evidence of malignancy, Fig. 3.

The patient was well in the post-operative period and he was discharged home with no post-operative complications.

### 3.2 Case 2

A 35-year-old male presented with chronic abdominal pain mainly in the right lower quadrant, the patient had non-relevant past medical and surgical histories. Abdominal examination showed tenderness on deep palpation at the right iliac fossa, abdominal ultrasound and abdominal X-ray were normal.

**Fig. 4:**
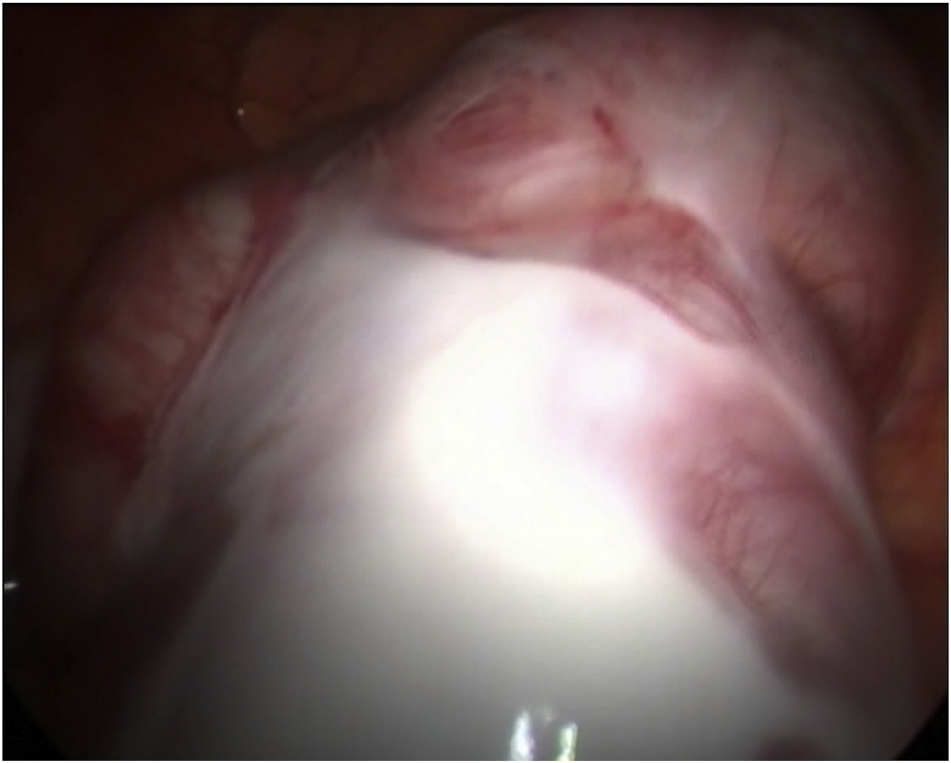
A laparoscopic picture showing the terminal ileum is enclosed by a thick layer with dilated proximal bowel loops.

During diagnostic laparoscopy the terminal ileum was found to be enclosed by a whitish thick membrane with dilated proximal segment of the ileum. Fig. 4.

**Fig. 5:**
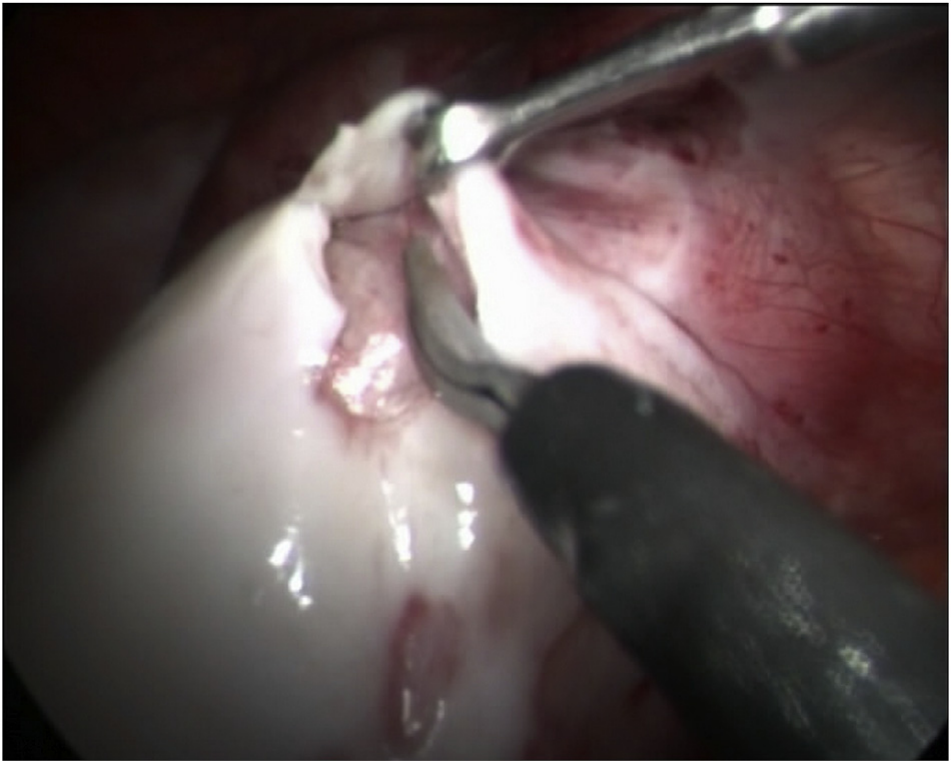
A laparoscopic picture showing the removal of the membrane over the bowel loops.

Release of the adhesions were done and biopsy was taken from the membranes which showed features of chronic inflammatory process with no evidence of malignancy. Fig. 5.

The patient was well in the post-operative period and he was discharged home with no post-operative complications.

## 4 Discussion

Abdomen cocoon should be differentiated from peritoneal encapsulation which was first described in 1868 because the etiology and the managements are different. In peritoneal encapsulation the bowel lies behind a secondary but normal peritoneal layer which is attached to the ascending, descending, and transverse colon. This bowel enters this sac from two sites; at the duodeno-jejunal and the ileocecal junctions. This condition is probably embryogenic in origin and the sac derived from the yolk sac, it may be associated with some other mesenteric abnormalities and most patients have no or mild symptoms in the form of abdominal pain and most cases discovered at autopsy. In the contrary cases of abdomen cocoon are symptomatic most of them requires surgical intervention due to intestinal obstruction, and its etiology remains unclear [3].

Tuberculous infection of the peritoneal cavity has been shown to cause a similar clinical picture, but the adhesions and the membrane are thicker and denser, although the manifestations of intra-peritoneal tuberculosis may be seen in some cases like mesenteric abscess formation, mesenteric lymph nodes enlargement and caseation, for this reason some authors advocate that this may be caused by tuberculous peritoneal infection [6,10,11].

Yip and Lee mentioned four clinical findings which may help in predicting the diagnosis before surgery, these findings are: occurrence of this presentation in young women, history of some similar attacks of the same pain which resolved spontaneously, repeated episodes of vomiting, and the presence of palpable soft and mobile abdominal pain, but these features are not specific and may present in a wide range of intra-abdominal pathologies [7].

CT scan of the abdomen have been shown to be the most valuable radiological investigation, it shows dilated small bowel loops in the center of the abdomen, it also identify the fibrous membrane surrounding the bowel loops forming a sac like structure, there may be little amount of fluid collection in-between the bowel loops, CT scan also exclude other causes of intestinal obstruction [6].

The normal peritoneal layer is composed of a single mesothelial cells that is supported by a basement membrane, while the histopathological examination of the membrane covering the bowel in patients with abdomen cocoon shows proliferation of fibrous and connective tissues, inflammatory cells infiltration, with no evidence of foreign body granuloma or giant cell formation [12].

In most cases the treatment is surgical and is discovered during surgery, a high index of suspicion in needed to diagnose the condition before surgery which is not the usual situation. The surgery involves removing the membrane and releasing the adhesions, sometimes resection of part of the bowel will be done if the viability in questionable, the condition has a very low risk of recurrence if no coexisting condition is present [2,10,[13], [14], [15]].

In the future larger number of patients must be collected and their data analyzed regarding the possible causes, the clinical presentation, the complications, and the long term follow up. This may involve collecting data from many different parts of the world or performing a large meta analyses study.

## Ethical approval

No ethical committee approval was needed; consent have been taken from the patients to report their findings.

## Sources of funding

No source of funding other than the authors.

## Author contribution

Study design, data collection, and data analysis: Dr Ayad Ahmad Mohammed and Dr Sardar Hassan Arif.

Writing the manuscript: Dr Ayad Ahmad Mohammed.

Final approval of the manuscript: Dr Ayad Ahmad Mohammed and Dr Sardar Hassan Arif.

## Unique identifying number (UIN)

5080 Research registry.

https://www.researchregistry.com.

## Trial registry number

N/A.

## Guarantor

Dr Ayad Ahmad Mohammed.

## Informed consent

An informed consent is taken from the patients to report the case and the accompanying images.

## Provenance and peer review

Not commissioned, externally peer reviewed.

## Declaration of competing interest

No conflicts of interest present.
